# Secukinumab in refractory non-infectious anterior uveitis

**DOI:** 10.3389/fopht.2025.1491981

**Published:** 2025-02-27

**Authors:** Otto M. Olivas-Vergara, Inés Hernanz, Pablo E. Borges-Deniz, Fredeswinda Romero-Bueno, Olga Sanchez-Pernaute, Ester Carreño

**Affiliations:** ^1^ Rheumatology Department, Fundación Jiménez Díaz University Hospital, Madrid, Spain; ^2^ Ophthalmology Department, Fundación Jiménez Díaz University Hospital, Madrid, Spain

**Keywords:** secukinumab, anterior uveitis, uveitis, spondyloarthritis, psoriatic arthritis

## Abstract

**Background:**

Secukinumab is a monoclonal antibody that selectively neutralizes interleukin-17A and has shown efficacy in the treatment of psoriatic arthritis, psoriasis, and axial spondyloarthritis. Its use in non-anterior non-infectious uveitis is controversial, with evidence generally not supporting its effectiveness in these conditions. However, the role of secukinumab in anterior non-infectious uveitis remains unclear.

**Methods:**

Case series. Five patients with biological therapy-refractory non-infectious anterior uveitis who were treated with secukinumab were included.

**Results:**

All 5 patients experienced a uveitis flare-up during treatment, and secukinumab failed to induce long-term remission in 2 of these patients, who also had uncontrolled systemic disease.

**Conclusion:**

Secukinumab failed to prevent uveitis flare-up in these patients with biological therapy-refractory disease. Further studies are necessary to determine the potential role of secukinumab in the treatment of anterior uveitis.

## Introduction

Secukinumab is a fully human monoclonal antibody that binds to and neutralizes interleukin-17A (IL-17A) with high affinity. Its effectiveness has been established for the treatment of psoriatic arthritis (PsA), psoriasis, and radiographic axial spondyloarthritis (Ax-SpA) (1). Despite the fact that non-infectious uveitis shares the same cytokine pathway with these inflammatory diseases in terms of their immunopathogenesis (1, 2), clinical trials using subcutaneous doses of secukinumab for non-infectious non-anterior uveitis have not shown significant differences in clinical outcomes compared to placebo. This has been observed in three different clinical trials (SHIELD, INSURE, and ENDURE) (3). Although a small trial with a high intravenous dose of secukinumab has shown positive results, it has not yet been validated in a larger cohort (4).

On the other hand, the effectiveness of secukinumab has not been evaluated in randomized clinical trials for non-infectious anterior uveitis. To date, no monoclonal antibody has been approved specifically for isolated anterior uveitis, although there is evidence that adalimumab, when used for systemic indications, can help control flare-ups of anterior uveitis (5, 6). Long-term monitoring of patients treated with secukinumab, who were included in pivotal clinical trials for Ax-SpA and a Swedish national database, has shown a low incidence of anterior uveitis. However, this incidence is higher when compared to monoclonal tumor necrosis factor inhibitors (TNFi) (7, 8). In the absence of clinical trials, reports on the outcomes of secukinumab treatment for non-infectious anterior uveitis can assist clinicians in determining the potential usefulness of this monoclonal antibody for this indication. The aim of this study is to report the real-world experience of the use of secukinumab in anterior uveitis.

## Methods

A retrospective review was conducted on the medical records of patients with anterior uveitis who were treated with secukinumab at Fundación Jiménez Díaz Hospital between 2017 and 2024. This study protocol was approved by the institutional review board of Fundacion Jimenez Diaz University Hospital (EOH018-20_FJD).

The decision of starting secukinumab was based in treating clinician’s criteria and the lack of response to conventional DMARDs (disease-modifying anti-rheumatic drugs) and at least one TNFi. Before starting secukinumab, there was not specific washout period. Secukinumab was started at the time of the next dose of the previous treatment. The patients with anterior uveitis were given secukinumab in the standard dose for PsA and Ax-SpA, which is 150mg subcutaneously every week for five weeks, followed by monthly maintenance dosing. If there was no clinical response based on the clinician’s criteria, the dose was increased to 300mg in accordance with the summary of product characteristics. Before administering the first dose of secukinumab, we followed national safety recommendations for the use of monoclonal antibodies and screened for active HIV, hepatitis B, hepatitis C, and tuberculosis (9).

For all patients, the following data were collected: age, gender, anatomical classification of the uveitis based on the criteria of the SUN working group (10), clinical phenotype, associated systemic disease, time from diagnosis to secukinumab treatment, previous treatment, treatment regimen at baseline, secukinumab dose, duration of treatment with secukinumab, and length of follow-up period. Systemic disease activity/control was based on treating-physician opinion.

The outcome measures of this study included the time to the first uveitis flare-up, time to the second uveitis flare-up, total number of uveitis flare-ups, and systemic disease control. The response to the treatment was evaluated on an individual basis. All the data collected were compiled into a database designed in Microsoft Excel 365 for retrospective analysis.

The SUN working group grading for anterior chamber inflammation (AC) was used (10). A flare-up of anterior uveitis was defined as the presence of at least 1+ AC cells in a previously quiet eye (≤0.5 + AC cells). All the anterior uveitis flare-ups were treated with a tapering regimen of topical dexamethasone or prednisolone acetate, starting with administration every 2 hours, as per local protocols. In case of macular edema associated to the anterior uveitis flare-up every 6 hours topical ketorolac (5mg/ml) was added to the topical steroids.

## Results

Five patients were identified (3 females and 2 male) with a mean age of 43 years (range 35-53 years) at the time of starting secukinumab treatment. The mean time of treatment duration after the first dose of secukinumab was 12.6 months (range 6-33 months).

All patients were diagnosed with non-infectious anterior uveitis and psoriasis or spondyloarthropathy-spectrum disease (PsA/Ax-SpA) as a systemic disease. Prior to receiving secukinumab, they had all been treated with at least one biologic DMARDs. Three out of 5 patients also used at least two conventional synthetic DMARDs previously. The decision to initiate secukinumab was based on active ocular or systemic disease and a lack of response or intolerance to biological therapy (TNFi or anti IL23, see **Table 1**). It is also noteworthy that Patient 1 was HIV positive and receiving antiretroviral therapy with an undetectable viral load and normal CD4 count. No other signs of advanced disease were detected during follow-up in this patient. We do not consider the anterior uveitis as infectious in this patient by its clinical characteristics (bilateral and non-hypertensive).

**Table 1:** Clinical characteristics and outcomes of patients with non-infectious anterior uveitis treated with secukinumab.

Patient	Age at diagnosis	Phenotypic classification	Associated systemic disease	HLAB-27 status	Previous IMT treatment	Treatment at baseline (added to secukinumab)	Time from diagnosis to secukinumab treatment	Time to first flare-up	Number of uveitic flare-ups	Duration of treatment with secukinumab
1	44	Acute recurrent	Ankylosing spondylitis, HIV	Positive	Sulfasalazine, methotrexate, cyclosporine, infliximab, adalimumab, golimumab	Methotrexate	9 years	12 weeks	3	6 months
2	35	Acute recurrent	Psoriatic arthritis	Negative	Methotrexate, sulfasalazine, certolizumab pegol	Methotrexate	5 years	16 weeks	2	8 months
3	36	Acute recurrent	Psoriatic arthritis	Positive	Methotrexate, sulfasalazine, adalimumab	Methotrexate	<1 year	28 weeks	1	12 months
4	34	Acute recurrent	Psoriasis	Negative	Ustekinumab	None	13 years	76 weeks	1	33 months
5	41	Acute recurrent	Non-radiographic spondyloarthritis	Positive	Adalimumab	None	1 year	4 weeks	1	4 months

Patients 1, 2 and 5 had their secukinumab dose increased to 300mg after 3, 8 and 4 months of treatment, respectively. Patient 3, on the other hand, increased to the 300mg dose due to uncontrolled systemic disease prior to experiencing the first episode of anterior uveitis. Patient 4 started with a dose of 300mg because of severe plaque psoriasis.

Patient 1 experienced a flare-up of uveitis 12 weeks after starting treatment with the 150mg dose of secukinumab and had a total of three flare-ups during follow-up. Despite increasing the dose to 300mg and continuing concomitant methotrexate therapy, active arthritis persisted, prompting the addition of off-label tofacitinib after six months of secukinumab treatment.

Patient 2 had a longer time to first flare-up, at 16 weeks after starting the 150mg dose of secukinumab but had two flare-ups during follow-up. Despite increasing the dose to 300mg and continuing concomitant methotrexate therapy, active arthritis persisted, and the patient refused further changes in therapy, including intraarticular steroid injections.

Patient 3 was diagnosed of non-infectious anterior uveitis after 28 weeks of starting treatment with secukinumab, but not presented new flares during the follow-up. In the same way, patient 4 presented a flare after 76 weeks of treatment.

Patient 5 experienced a flare after 4 weeks of treatment and the dose was increased from 150mg to 300mg monthly, but until last visit she still continued with inflammatory back pain without new flares.

No adverse events were recorded during treatment in our patients.

## Discussion

In this study, the clinical response to secukinumab was evaluated in five patients with anterior non-infectious uveitis refractory to biological therapy. Despite receiving the maximum dose of 300mg per month, all five patients experienced uveitis flare-ups during treatment. Three of the patients also had a clinically uncontrolled systemic disease (PsA/Ax-SpA), and secukinumab failed to induce a long-lasting remission in these cases, even though there has not been a specific washout period for the previous biological therapy. The study’s findings are in line with previous clinical trials that evaluated the use of secukinumab for non-anterior uveitis and found it to be ineffective. We acknowledge that our study has several limitations: small sample, different systemic diseases, systemic disease control based on treating-physician opinion and not in a specific disease index, and lack of specific washout period. Despite these limitations, our study resembles a real-life scenario where there is no data from clinical trials.

The study by Miserocchi et al. showed a positive response to secukinumab in patients with HLA-B27 associated uveitis, which is a more homogeneous sample than the patients in our study (11). It is important to note that the patients in both studies were refractory to conventional immunosuppressors and TNFi, making them a difficult-to-treat subset. Additionally, the sample size was small. It is possible that refractory patients may benefit from intravenous administration of secukinumab, although this approach has not been tested in isolated non-infectious anterior uveitis and remains speculative.

The attempt to develop secukinumab for non-anterior uveitis parallels its unsuccessful trials in rheumatoid arthritis (RA). In RA, IL-17 is overexpressed in animal models and in patient samples, but clinical trials, including those that tested intravenous secukinumab, failed to show significant clinical benefit (12). This may be due to the highly variable expression of IL-17A, IL-17F, and their receptors among individual patients at the tissue level (13). Identifying patients who are more likely to respond to anti-IL 17 treatment based on tissular IL-17 expression may be possible, but it is not yet feasible in ophthalmological clinical practice due to the associated risk of obtaining intraocular samples.

A recent approved drug for axial spondyloarthritis, bimekizumab, a monoclonal antibody that selectively inhibits IL-17A and IL-17F, has showed a lower incidence rate of uveitis among patients randomized to this drug in a pooled analysis from phase 2b/3 trials (14). Although these results should be taken with caution because of the limitations of this study (low overall rate of events, short duration of treatment period, events not assessed by an ophthalmologist), this clinical data suggests that inhibition of IL-17A and IL-17F may be effective for the prevention of non-infectious anterior uveitis in this scenario (14).

In conclusion, secukinumab failed to prevent uveitis flare-up in these patients with biological therapy-refractory disease. Further research is needed to understand the role of IL-17 inhibitors in anterior and non-anterior uveitis.

## Data Availability

The original contributions presented in the study are included in the article/supplementary material. Further inquiries can be directed to the corresponding author.
